# Diagnostic value of pentraxin 3 in plasma and bronchoalveolar lavage fluid for invasive pulmonary aspergillosis in non-neutropenic patients: a prospective multicenter clinical study

**DOI:** 10.1080/22221751.2026.2667562

**Published:** 2026-04-29

**Authors:** Chao Sun, Huanhuan Zhong, Xiaomin Cai, Min Cao, Tingting Zhao, Li Wang, Chunlai Feng, Wenkui Sun, Cheng Chen, Yanbin Chen, Yujian Tao, Jun Zhou, Yueyan Ni, Yu Gu, Minhua Shi, Xin Lu, Guoer Ma, Yuanqin Li, Jiaxin Shi, Yuchen Cai, Mengyue Song, Yuanyuan Li, Yajie Lu, Jinjin Zhong, Yi Shi, Xin Su

**Affiliations:** a Department of Respiratory and Critical Medicine, Nanjing Drum Tower Hospital, Affiliated Hospital of Medical School, Nanjing University, Nanjing, People’s Republic of China; b Department of Respiratory and Critical Medicine, Jinling Hospital, Affiliated Hospital of Medical School, Nanjing University, Nanjing, People’s Republic of China; c Department of Respiratory and Critical Medicine, the Second Affiliated Hospital of Soochow University, Suzhou, People’s Republic of China; d Department of Respiratory and Critical Medicine, Nanjing First Hospital, Nanjing, People’s Republic of China; e Department of Respiratory and Critical Medicine, Jiangsu Province Second Chinese Medicine Hospital, Nanjing, People’s Republic of China; f Department of Respiratory and Critical Medicine, Changzhou First People’s Hospital, Changzhou, People’s Republic of China; g Department of Respiratory and Critical Medicine, Jiangsu Province Hospital, Nanjing, People’s Republic of China; h Department of Respiratory and Critical Medicine, the First Affiliated Hospital of Soochow University, Suzhou, People’s Republic of China; i Department of Respiratory and Critical Medicine, Affiliated Hospital of Yangzhou University, Yangzhou, People’s Republic of China; j Department of Respiratory and Critical Medicine, Nanjing Jiangning Hospital, Nanjing, People’s Republic of China; k Department of Respiratory and Critical Medicine, Affiliated Hospital of Jiangsu University, Zhenjiang, People’s Republic of China; l Department of Respiratory and Critical Medicine, Affiliated Hospital of Xuzhou Medical University, Xuzhou, People’s Republic of China

**Keywords:** Pentraxin 3, invasive pulmonary aspergillosis, non-neutropenic patients, diagnosis, biomarker

## Abstract

To evaluate the diagnostic performance of pentraxin 3 (PTX3) in plasma and bronchoalveolar lavage fluid (BALF) for invasive pulmonary aspergillosis (IPA) in non-neutropenic patients. This prospective, multicenter cohort study enrolled 605 patients with clinically suspected IPA, including 195 IPA cases and 410 non-IPA controls. BALF and/or plasma samples were collected from all participants. PTX3 levels were measured by enzyme-linked immunosorbent assay. Diagnostic value was assessed using receiver operating characteristic curve analysis. PTX3 levels in both BALF and plasma were significantly higher in IPA patients than in non-IPA controls (BALF: 8.98 [3.47–22.53] ng/ml vs. 1.12 [0.46–3.35] ng/ml, *P* < 0.0001; plasma: 8.29 [4.68–21.41] ng/ml vs. 2.60 [1.35–5.02] ng/ml, *P* < 0.0001). The optimal cutoff value of BALF PTX3 for diagnosing IPA was 2.67 ng/ml, with an area under the curve (AUC) of 0.84 (95% CI, 0.80–0.89), sensitivity of 85.57% (95% CI, 77.22–91.20), and specificity of 71.72% (95% CI, 66.28–76.60). For plasma PTX3, the optimal cutoff value was 4.17 ng/ml, with an AUC of 0.80 (95% CI, 0.75–0.85), sensitivity of 79.51% (95% CI, 71.50–85.72), and specificity of 69.79% (95% CI, 64.26–74.81). In paired analysis, BALF PTX3 showed significantly higher sensitivity than BALF galactomannan (GM) (86.96% vs. 72.83%, *P* = 0.037). Parallel combination of BALF PTX3 and GM increased diagnostic sensitivity to 97.83%. BALF and plasma PTX3 are valuable adjunctive biomarkers for non-neutropenic IPA patients, especially when combined with GM for early IPA rule-out.

**Clinical trial registration:**

ClinicalTrials.gov (registration number: ChiCTR2000039235).

## Introduction

Invasive pulmonary aspergillosis (IPA) is a life-threatening fungal infection which predominantly affects immunocompromised individuals. In recent years, a notable rise in IPA incidence has been observed among non-neutropenic patients, largely attributable to the widespread administration of corticosteroids, the high prevalence of chronic obstructive pulmonary disease (COPD), and advancements in intensive care practices. Globally, over 2.1 million cases of invasive aspergillosis were reported annually, underscoring its substantial global burden [1]. However, the clinical presentation and radiological features of IPA in non-neutropenic patients are often nonspecific, making early diagnosis challenging. Currently, the definitive diagnosis of IPA relies on histopathological confirmation [2], which is invasive and impractical for routine clinical application. Traditional fungal cultures of respiratory specimens are time-consuming and have low sensitivity. Although galactomannan (GM) detection in bronchoalveolar lavage fluid (BALF) is more sensitive than in plasma, its overall sensitivity remains suboptimal [3]. Metagenomic next-generation sequencing (mNGS) offers high sensitivity but cannot reliably distinguish *Aspergillus* colonization from true infection [4]. Thus, reliable and rapid biomarkers are urgently needed to facilitate early and accurate diagnosis of IPA in non-neutropenic patients.

Pentraxin 3 (PTX3) is a soluble pattern recognition receptor rapidly synthesized at inflammation sites and plays an essential role in innate immune response [5]. Increasing evidence has highlighted the critical role of PTX3 in anti-*Aspergillus* immunity [6]. Mechanistically, PTX3 recognizes *Aspergillus fumigatus* conidia and promotes phagocytosis by neutrophils and macrophages through interactions with the complement system and Fcγ receptors [7,8]. PTX3 deficiency in mice leads to increased susceptibility to *Aspergillus* infection [9]. Furthermore, specific PTX3 gene polymorphisms have been associated with increased susceptibility to IPA, further confirming its indispensable function in host defense against *Aspergillus* [10–14]. Given its rapid local production in response to *Aspergillus* invasion, PTX3 has emerged as a promising host-derived diagnostic biomarker for pulmonary aspergillosis (PA). Our preliminary single-centre study demonstrated elevated PTX3 levels in patients with PA [15]. However, its diagnostic utility was constrained by cohort heterogeneous and the limited sample size of the IPA subset. Although subsequent investigations have assessed PTX3 diagnostic utility in specific subpopulations, such as COPD patients complicated by IPA [16], extensive multicenter validation remains critically needed. Establishing standardized diagnostic thresholds and clinical protocols for PTX3 application in non-neutropenic patients at risk for IPA is essential to fully realize its potential as a diagnostic tool.

This study aims to elucidate the diagnostic value of PTX3 in non-neutropenic patients with IPA. We measured PTX3 levels in BALF and plasma samples between IPA and non-IPA patients and compared its diagnostic value with traditional mycological biomarkers. The results are expected to provide new insights for the early diagnosis of non-neutropenic IPA, potentially advancing diagnostic strategies and improving clinical outcomes.

## Methods

### Study design

This prospective multicenter cohort study was conducted at 12 hospitals in Jiangsu, China, from August 2020 to February 2024. Eligible patients met the following criteria: (i) age ≥18 years; (ii) hospitalization with suspected IPA; and (iii) provision of written informed consent. Suspected IPA was defined by the presence of: (i) host factors, including chronic pulmonary or extrapulmonary disease, such as COPD, diabetes or intensive care unit (ICU) admission; (ii) clinical symptoms, such as respiratory manifestations including cough, sputum, dyspnea, or chest pain that were unresponsive to broad-spectrum antibiotics; and (iii) abnormal chest computed tomography (CT) findings, such as nodules, cavitation, or consolidation. Exclusion criteria were: (i) neutropenia during hospitalization (<0.5 × 10^9^/L); (ii) uncertain final diagnosis; (iii) incomplete clinical data; and (iv) prior antifungal therapy before sample collection. BALF and/or peripheral blood samples were collected from all patients.

The diagnosis of IPA was established based on the 2020 European Organization for Research and Treatment of Cancer and Mycoses Study Group Education and Research Consortium (EORTC/MSGERC) consensus definitions [2] and the 2024 criteria from ESGCIP, EFISG, ESICM, ECMM, MSGERC, ISAC, and ISHAM [17]. Detailed diagnostic criteria are provided in the Supplementary Methods.

The study was approved by the Ethics Committee of the Jinling Hospital (2020NZKY-012-01). Written informed consent was obtained from all participants.

### Sample size estimation

In our previous single-centre study of non-neutropenic populations, plasma PTX3 demonstrated an area under curve (AUC) of 0.854 for diagnosing PA in 36 PA patients (including both invasive and chronic pulmonary aspergillosis) and 137 COPD patients. For diagnosing IPA, the AUC was 0.915 in a subset of 25 IPA patients and 137 COPD patients [10]. In a subsequent study, 107 PA patients (51 BALF and 89 plasma samples) and 324 non-PA patients (160 BALF and 218 plasma samples) were included. The AUC for BALF PTX3 in diagnosing PA was 0.91, while the AUC for plasma PTX3 was 0.84 [15]. Therefore, based on these findings, we expect the diagnostic AUC of PTX3 for IPA to reach 0.88. Assuming a null hypothesis AUC of no less than 0.80, with an expected proportion of IPA cases at 1/3 among the enrolled population, a two-sided significance level of 0.05, and a power of 80%, a total of 77 IPA cases and 203 non-IPA cases (307 in total) would be required. Considering a 10% dropout or loss to follow-up, this diagnostic trial aims to enrol 342 patients. The sample size estimation was performed using PASS version 11.

### Patients and samples

**Figure 1.**
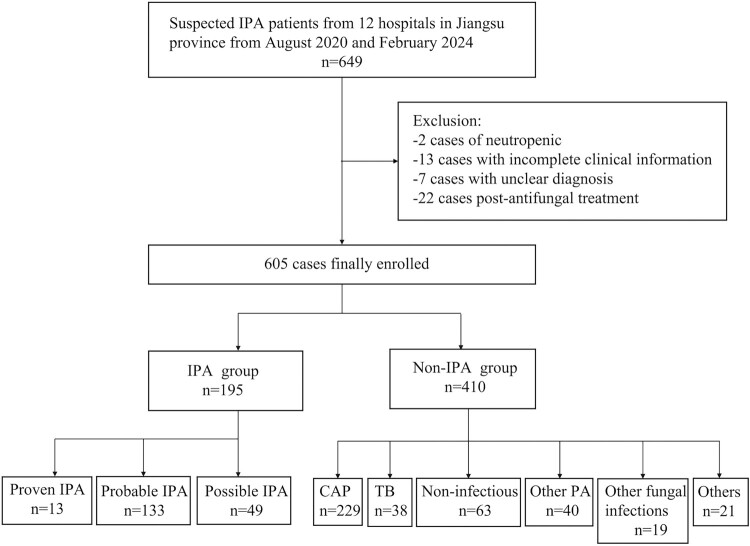
The flowchart of study. IPA, invasive pulmonary aspergillosis; CAP, community-acquired pneumonia; TB, tuberculosis; PA, pulmonary aspergillosis.

The study flowchart is shown in Figure 1. A total of 649 patients with suspected IPA were initially enrolled, of whom 44 were excluded. Consequently, 605 patients were included in the final analysis, comprising 411 BALF samples and 445 peripheral blood samples. According to the diagnostic criteria, 195 patients were diagnosed with IPA, including 13 proven cases, 133 probable cases, and 49 possible cases. Only proven and probable IPA cases were included in the final analysis. 97 BALF and 122 peripheral blood samples were obtained from IPA patients (proven and probable cases). The non-IPA group consisted of 410 patients, with 290 BALF samples and 288 peripheral blood samples. These patients were diagnosed with community-acquired pneumonia (CAP) (*n* = 229), tuberculosis (TB) (*n* = 38), nontuberculous mycobacteriosis (*n* = 11), other types of PA (*n* = 40), other fungal infections (*n* = 19), lung abscess (*n* = 10), and non-infectious diseases (*n* = 63). Additionally, 70 peripheral blood samples were collected from healthy controls.

### PTX3 measurement and data collection

BALF and peripheral blood samples from each centre were transported to the central laboratory under cold chain conditions. All samples were coded by a dedicated researcher and processed for centralized testing. Laboratory technicians were fully blinded to the patients’ clinical information and final diagnoses. PTX3 concentrations were measured with a commercial enzyme-linked immunosorbent assay (ELISA) kit (DPTX30, Quantikine Human Pentraxin 3 Immunoassay, R&D Systems, USA). Plasma *Aspergillus*-specific IgG antibody (*Asp* IgG) levels were assessed using a quantitative test kit (Dynamiker, China). Case report forms were completed at each centre to record demographic data, radiological findings, and mycological results.

### Statistics

Statistical analyzes were performed using SPSS (version 29.0, IBM Corporation, NY, USA). Categorical variables were expressed as frequencies or percentages and compared using the chi-square test or Fisher's exact test. Continuous variables were expressed as mean ± standard deviation (SD) or median (interquartile range, IQR) and analyzed using the independent Students' *t* test or Mann–Whitney *U* test (two groups) and one-way ANOVA or Kruskal–Wallis H test (multiple groups), as appropriate. Diagnostic value was evaluated by receiver operating characteristic (ROC) curve analysis, with optimal cutoff value determined by the Youden index. McNemars’ test was used to compare the sensitivity and specificity of paired binary diagnostic results. The DeLong test was utilized to compare the differences between the areas under the ROC curves (AUCs). A two-tailed *P* value <0.05 was considered statistically significant.

## Results

### Patient characteristics

The clinical characteristics of IPA and non-IPA patients are shown in Table 1. Compared with non-IPA patients, IPA patients had a significantly higher prevalence of COPD (*P < *0.05). Chest CT scans revealed more frequent cavitation, tree-in-bud signs, and pleural effusion in the IPA group (*P < *0.05). Additionally, IPA patients were older and had a higher rate of ICU admissions (*P < *0.05).

**Table 1.** Clinical characteristics of IPA and non-IPA patients.

	BALF			Plasma		
	IPA (*n *= 97)	non-IPA (*n *= 290)	*P*	IPA (*n *= 122)	non-IPA (*n *= 288)	*P*
Age, year, media [IQR]	69 [59, 76]	60 [53, 69]	*<*0.0001	69 [63, 75]	63 [55, 71]	<0.0001
Male, *n* (%)	73 (75.26)	191 (65.86)	0.085	96 (78.69)	202 (70.14)	0.077
Host factors, *n* (%)						
Chronic lung diseases						
COPD	30 (30.93)	24 (8.28)	<0.0001	35 (28.69)	38 (13.19)	0.0002
Lung cancer	11 (11.34)	27 (9.31)	0.561	17 (13.93)	28 (9.72)	0.212
Bronchiectasis	24 (24.74)	60 (20.69)	0.402	25 (20.49)	62 (21.53)	0.815
Extrapulmonary diseases						
diabetes	27 (27.84)	54 (18.62)	0.054	30 (24.59)	68 (23.94)	0.889
Solid tumour	19 (19.59)	34 (11.72)	0.051	25 (20.49)	38 (13.19)	0.061
Autoimmune disease	8 (8.25)	45 (15.52)	0.071	11 (9.02)	28 (9.72)	0.824
Medical history						
Systemic glucocorticoids^a^	22 (22.68)	46 (15.86)	0.127	29 (23.77)	48 (16.67)	0.092
Immunosuppressants^b^	5 (5.15)	33 (11.38)	0.075	4 (3.28)	23 (7.99)	0.079
Chest CT features, *n* (%)						
Nodules						
1–3 cm in diameter	33 (34.02)	84 (28.97)	0.348	36 (29.51)	82 (28.47)	0.832
<1 cm in diameter	42 (43.30)	111 (38.28)	0.381	42 (34.43)	105 (36.46)	0.695
Cavitation	32 (32.99)	50 (17.24)	0.001	35 (28.69)	52 (18.06)	0.016
Air-crescent sign	4 (4.12)	4 (1.38)	0.112	5 (4.10)	3 (1.04)	0.054
Tree-in-bud pattern	8 (8.25)	4 (1.38)	0.002	8 (6.56)	2 (0.69)	0.001
Pleural effusion	45 (46.39)	70 (24.14)	<0.0001	62 (50.82)	72 (25.00)	<0.0001
Severity						
ICU admission, *n* (%)	45 (46.39)	50 (17.24)	<0.0001	66 (54.10)	62 (21.53)	<0.0001

BALF, bronchoalveolar lavage fluid; IPA, invasive pulmonary aspergillosis; IQR, interquartile range; COPD, chronic obstructive pulmonary disease; CT, computed tomography; ICU, intensive care unit.

^a^ Oral or intravenous glucocorticoids were used ≥20 mg/day for more than 3 weeks within a 60-day period;

^b^ Immunosuppressive drugs were used within a 30-day period.

### PTX3 levels in BALF

**Figure 2.**
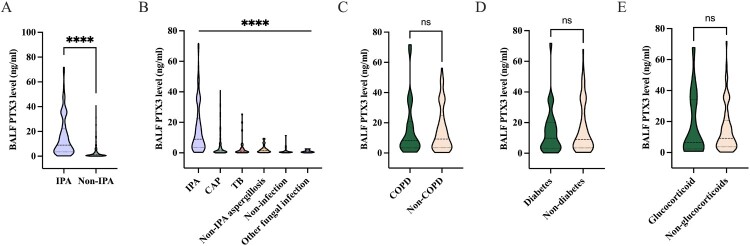
Comparative analysis of BALF PTX3 levels across various groups. (A) BALF PTX3 levels in IPA group, and non-IPA group. (B) BALF PTX3 levels among patients with IPA, CAP, TB, other types of PA, non-infectious diseases, and other fungal infections. (C) BALF PTX3 levels among IPA patients with or without COPD. (D) BALF PTX3 levels among IPA patients with or without diabetes. (E) BALF PTX3 levels among IPA patients who used or did not use corticosteroids. ****P < 0.0001; ns: *P *> 0.05. BALF, bronchoalveolar lavage fluid; IPA, invasive pulmonary aspergillosis; CAP, community-acquired pneumonia; TB, tuberculosis; PA, pulmonary aspergillosis.

The median BALF PTX3 level was significantly elevated in IPA group compared to non-IPA group (8.98 [3.47–22.53] ng/ml vs. 1.12 [0.46–3.35] ng/ml, *P* < 0.0001; Figure 2(A)). When non-IPA patients were categorized into CAP (1.26 [0.46–4.48] ng/ml), TB (1.14 [0.44–3.04] ng/ml), other types of PA (1.59 [0.53–2.88] ng/ml), non-infectious diseases (0.48 [0.29–1.00] ng/ml), and other fungal infections (0.74 [0.35–1.77] ng/ml) groups, the BALF PTX3 level in IPA group remained significantly higher than in each subgroup (all *P* < 0.05; Figure 2(B), Table S1). Further comparisons showed no significant differences in BALF PTX3 levels among IPA patients with or without COPD (8.31 [3.41–20.62] ng/ml vs. 9.23 [3.43–24.71] ng/ml, *P* = 0.795), diabetes (9.85 [3.32–19.96] ng/ml vs. 8.98 [3.51–26.21] ng/ml, *P* = 0.719), or systemic corticosteroid use (6.52 [2.37–34.05] ng/ml vs. 9.22 [3.70–20.83] ng/ml, *P *= 0.682; Figure 2(C–E)). Moreover, BALF PTX3 levels were comparable between patients with positive and negative results for BALF GM (11.65 [3.70–34.24] ng/ml vs. 6.94 [3.07–13.92] ng/ml, *P* = 0.120) and plasma GM (13.17 [5.70–30.51] ng/ml vs. 8.15 [2.93–19.54] ng/ml, *P* = 0.131; Figure S1).

### PTX3 levels in plasma

**Figure 3.**
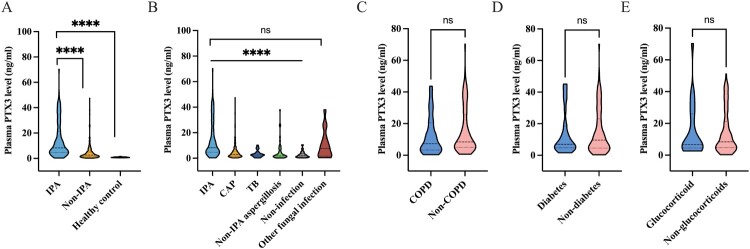
Comparative analysis of plasma PTX3 levels across different groups. (A) Plasma PTX3 levels in IPA group, non-IPA group, and healthy cohort. (B) Plasma PTX3 levels among patients with IPA, CAP, TB, other types of PA, non-infectious diseases, and other fungal infections. (C) Plasma PTX3 levels among IPA patients with or without COPD. (D) Plasma PTX3 levels among IPA patients with or without diabetes. (E) Plasma PTX3 levels among IPA patients who used or did not use corticosteroids. ****P < 0.0001. IPA, invasive pulmonary aspergillosis; CAP, community-acquired pneumonia; TB, tuberculosis; PA, pulmonary aspergillosis.

Plasma PTX3 levels were significantly higher in IPA patients than in non-IPA patients (8.29 [4.68–21.41] ng/ml vs. 2.60 [1.35–5.02] ng/ml, *P* < 0.0001) and healthy controls (0.82 [0.66–1.06] ng/ml, *P* < 0.0001; Figure 3(A)). Compared with CAP (2.91 [1.62–5.41] ng/ml), TB (2.65 [1.47–4.28] ng/ml), other types of PA (1.87 [0.86–3.50] ng/ml), and non-infectious diseases (2.09 [1.00–3.70] ng/ml), plasma PTX3 levels remained significantly higher in IPA patients (all *P* < 0.05; Figure 3(B), Table S1). However, no significant difference was observed between IPA and those with other fungal infections (7.62 [1.59–15.75] ng/ml, *P* = 0.175; Figure S2). Additionally, there was no difference in plasma PTX3 level between patients with pulmonary mucormycosis and IPA (11.51 [6.66, 25.74], *P* = 0.526). Stratification by comorbidities also showed no significant differences in plasma PTX3 levels in IPA patients with or without COPD (7.29 [3.30–20.47] ng/ml vs. 8.44 [4.93–25.61] ng/ml, *P* = 0.232), diabetes (6.98 [4.79–18.39] ng/ml vs. 9.60 [4.59–23.10] ng/ml, *P* = 0.762), or prior systemic corticosteroid use (6.63 [4.52–25.96] ng/ml vs. 8.44 [4.74–21.20] ng/ml, *P* = 0.918; Figure 3(C–E)). Notably, plasma PTX3 levels showed no significant differences between groups stratified by positive or negative results for BALF GM (9.12 [4.54–20.47] ng/ml vs. 6.81 [4.74–25.73] ng/ml, *P* = 0.947) and plasma GM (11.70 [5.22–30.68] ng/ml vs. 6.63 [4.67–15.14] ng/ml, *P* = 0.055; Figure S3).

### Diagnostic value of PTX3 in BALF and plasma samples

**Figure 4.**
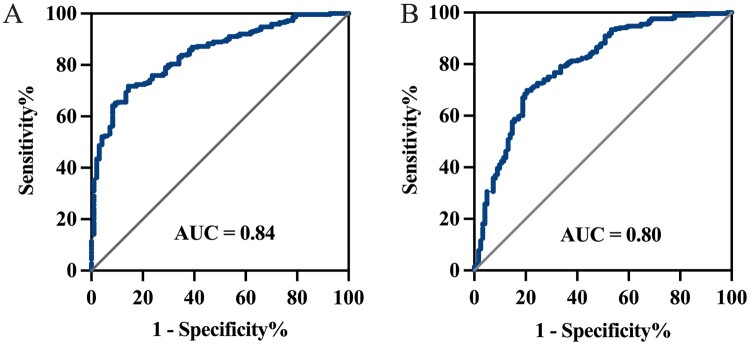
ROC curve analysis of BALF and plasma PTX3 for the diagnosis of IPA. (A) ROC curve analysis of BALF PTX3 for the diagnosis of IPA. (B) ROC curve analysis of plasma PTX3 for the diagnosis of IPA. ROC, receiver operating characteristic; PTX3, pentraxin 3; IPA, invasive pulmonary aspergillosis; BALF, bronchoalveolar lavage fluid; AUC, area under curve.

ROC analysis identified a BALF PTX3 cutoff value of 2.67 ng/ml for IPA diagnosis, with an AUC of 0.84 (95% CI, 0.80–0.89), sensitivity of 85.57% (95% CI, 77.22–91.20), and specificity of 71.72% (95% CI, 66.28–76.60; Figure 4(A)). The optimal plasma PTX3 cutoff value was 4.17 ng/ml, yielding an AUC of 0.80 (95% CI, 0.75–0.85), sensitivity of 79.51% (95% CI, 71.50–85.72), and specificity of 69.79% (95% CI, 64.26–74.81; Figure 4(B)). The sensitivity and specificity were comparable between plasma and BALF PTX3 (*P* = 0.093 and *P* = 0.609, respectively).

### Sensitivity analysis

To evaluate the impact of excluding possible IPA cases on our primary diagnostic models, we conducted a sensitivity analysis incorporating the 49 initially excluded possible IPA cases. First, we observed a significant stepwise expression of PTX3 across different diagnostic levels. For BALF PTX3, the median levels in the possible IPA group (3.84 [0.94–7.73] ng/ml) were significantly lower than those in the proven/probable IPA group (8.98 [3.47–22.53] ng/ml, *P < *0.001), but significantly higher than in the non-IPA group (1.12 [0.46–3.35] ng/ml, *P* = 0.001). A similar intermediate pattern was observed for plasma PTX3 (possible IPA vs. proven/probable IPA: 4.65 [1.95–9.23] vs. 8.29 [4.68–21.41] ng/ml, *P* = 0.003; possible IPA vs. non-IPA: 2.60 [1.35–5.02] ng/ml, *P* = 0.005; Figure S4).

Furthermore, we recalculated the diagnostic performance by reclassifying the possible IPA cases. When possible IPA cases were assumed to be IPA group, the AUCs for BALF and plasma PTX3 slightly decreased to 0.81 (95% CI, 0.77–0.85) and 0.76 (95% CI, 0.71–0.81), respectively. Conversely, classifying them as non-IPA group yielded AUCs of 0.83 (95% CI, 0.79–0.88, BALF) and 0.78 (95% CI, 0.73–0.83, plasma). Despite these expected mild fluctuations due to the inherent uncertainty of possible IPA cases, the overall diagnostic value of PTX3 remained robust. Detailed diagnostic metrics are provided in Table S2.

After adjusting for confounding factors (including age, ICU admission, COPD, diabetes, and co-existing bacterial infection), multivariable logistic regression model demonstrated that both BALF and plasma PTX3 remained independent predictors for the diagnosis of IPA. Specifically, the odds ratio (OR) for BALF PTX3 was 1.11 (95% CI, 1.07–1.16), and for plasma PTX3 was 1.07 (95% CI, 1.04–1.11; Table S3). Furthermore, inverse probability of treatment weighting (IPTW)-weighted ROC curve analysis revealed that the AUC of BALF PTX3 for diagnosing IPA was 0.82 (95% CI, 0.76–0.87), while the AUC of plasma PTX3 was 0.75 (95% CI, 0.69–0.81; Figure S5).

### Comparison of the diagnostic performance of PTX3 with other microbiological biomarkers

The diagnostic performance of PTX3 in comparison with other microbiological biomarkers is summarized in Table 2. Among the entire IPA cohort, the positive rates of sputum culture (50/131), BALF culture (24/104), plasma GM (48/139), BALF GM (93/126), BALF mNGS (55/74), and plasma *Asp* IgG (58/100) were 38.17%, 23.08%, 34.53%, 73.81%, 74.32% and 58.00%, respectively.

**Table 2.** Comparison of the diagnostic value of PTX3, GM, mNGS and *Asp* IgG in IPA patients.

	Cutoff value	Sensitivity % (95% CI)	Specificity % (95% CI)	PPV % (95% CI)	NPV % (95% CI)
BALF					
PTX3	2.67 ng/ml	85.57 (77.22–91.20)	71.72 (66.28–76.60)	50.30 (42.75–57.83)	93.69 (89.70–96.21)
GM	1.0 ODI	73.81 (65.51–80.70)	88.60 (84.56–91.69)	72.66 (64.31–79.67)	89.18 (85.17–92.21)
mNGS	Positive detection	74.32 (63.35–82.90)	80.00 (73.83–85.01)	58.51 (48.41–67.94)	89.14 (83.67–92.94)
PTX3/GM	–^c^	97.83 (92.42–99.40)	63.04 (56.98–68.70)	48.65 (41.55–55.81)	98.79 (96.66–99.66)
Plasma					
PTX3	4.17 ng/ml	79.51 (71.50–85.72)	69.79 (64.26–74.81)	52.72 (45.52–59.80)	88.94 (84.18–92.39)
GM	0.5 ODI	34.53 (26.89–43.06)	93.64 (90.64–95.74)	68.57 (57.09–78.16)	78.05 (73.79–81.72)
*Asp* IgG	80 AU/ml	58.00 (48.25–67.13)	66.53 (60.47–72.06)	40.85 (33.26–49.02)	79.90 (73.79–84.82)
PTX3/GM	–^c^	85.34 (77.62–90.79)	63.82 (57.60–69.59)	52.66 (45.09–60.11)	90.23 (84.96–93.76)
PTX3/GM/	–^c^	93.62 (86.79–97.12)	44.19 (37.68–50.91)	42.31 (35.66–49.27)	94.06 (87.91–97.26)
*Asp* IgG					

BALF, bronchoalveolar lavage fluid; PTX3, pentraxin 3; GM, galactomannan; ODI, optical density index; *Asp* IgG, *Aspergillus*-specific IgG antibody; IPA, invasive pulmonary aspergillosis; PPV, positive predictive value; NPV, negative predictive value.

^c^ A parallel testing strategy was used for combined biomarkers, whereby a positive result was defined as positivity in at least one component of the combination.

To rigorously evaluate diagnostic efficacy, pairwise comparisons were performed in matched cohorts. In 232 patients who underwent both BALF PTX3 and BALF mNGS testing (57 IPA, 175 non-IPA), the two assays showed comparable sensitivity (78.95% vs. 75.44%, *P* = 0.832) and specificity (70.86% vs. 78.29%, *P* = 0.144). In 349 patients with both BALF PTX3 and BALF GM data (92 IPA, 257 non-IPA), BALF PTX3 demonstrated significantly higher sensitivity than BALF GM (86.96% vs. 72.83%, *P* = 0.037) but significantly lower specificity (70.04% vs. 91.05%, *P* < 0.001). Similarly, in 301 patients with paired plasma PTX3 and BALF GM measurements (102 IPA, 199 non-IPA), plasma PTX3 showed nonsignificantly higher sensitivity than BALF GM (78.22% vs. 71.29%, *P* = 0.286) but significantly lower specificity (67.34% vs. 84.42%, *P* < 0.001). DeLong test showed BALF PTX3 and BALF GM had comparable overall diagnostic performance (AUC: 0.83 vs. 0.87, *P* = 0.275), both significantly outperforming plasma PTX3 (*AUC* = 0.75; *P* = 0.049 and *P* = 0.006, respectively).

Finally, a parallel testing strategy was applied, in which the test result was considered positive if any one of the combined markers yielded a positive result. Using this approach, combining BALF PTX3 with GM or plasma PTX3 with GM significantly improved diagnostic sensitivity, reaching 97.83% (95% CI, 92.42–99.40) and 85.34% (95% CI, 77.62–90.70), respectively. Furthermore, the combination of plasma PTX3, GM, and *Asp* IgG further enhanced sensitivity to 93.62% (95% CI, 86.79–97.12).

### Diagnostic value of PTX3 in ICU patients with IPA

**Figure 5.**
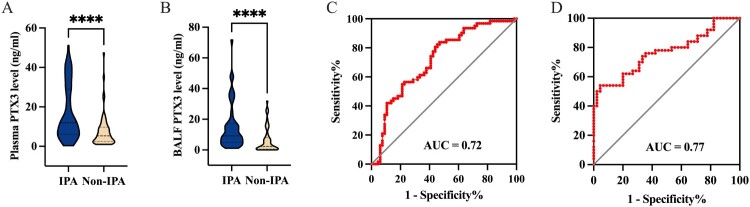
Levels and diagnostic performance of plasma and BALF PTX3 in IPA patients admitted to the ICU. (A,B) Levels of plasma and BALF PTX3 in IPA patients and non-IPA patients admitted to the ICU. (C-D) ROC curve analysis of plasma and BALF PTX3 for the diagnosis of IPA patients admitted to the ICU. ****P < 0.0001. PTX3, pentraxin 3; IPA, invasive pulmonary aspergillosis; BALF, bronchoalveolar lavage fluid; ICU, intensive care unit; ROC, receiver operating characteristic; AUC, area under curve.

Given previous findings associating PTX3 levels with the severity and prognosis of IPA, we further assessed its diagnostic utility in a subgroup of ICU patients [18]. In this cohort, both plasma and BALF PTX3 levels were significantly higher in the IPA group compared to non-IPA group (11.96 [6.10–29.01] ng/ml vs. 5.35 [2.45–9.66] ng/ml, *P* < 0.0001 for plasma; 9.22 [4.90–17.11] ng/ml vs. 2.21 [0.53–8.04] ng/ml, *P* < 0.0001 for BALF) (Figure 5(A,B)). Applying the overall cohort cutoff values (4.17 ng/ml for plasma and 2.67 ng/ml for BALF), plasma PTX3 achieved a sensitivity of 87.88% (95% CI, 77.86–93.73) and specificity of 41.94% (95% CI, 30.48–54.34), whereas BALF PTX3 reached a sensitivity of 95.56% (95% CI, 85,17–98.77) and specificity of 54.00% (95% CI, 40.40–67.03).

ROC analysis indicated that increasing the plasma PTX3 cutoff to 11.81 ng/ml improved specificity to 83.87% (95% CI, 72.79–91.00), but sensitivity decreased to 53.03% (95% CI, 41.16–64.57; Figure 5(C)). The optimal cutoff value for BALF PTX3 remained at 2.67 ng/ml (Figure 5(D)). The combined diagnostic performance of PTX3, GM, mNGS and *Asp* IgG in this subgroup was summarized in Table S4.

### Dynamic changes in PTX3 levels after antifungal treatment

Among 10 IPA survivors responsive to antifungal therapy, plasma PTX3 levels significantly declined after at least two weeks of therapy (*P* = 0.002; Figure S6).

## Discussion

The diagnosis of IPA currently relies predominantly on mycological biomarkers, which exhibit low sensitivity and fail to distinguish between *Aspergillus* colonization and infection. To address these limitations, our study evaluates the host immune response to *Aspergillus* by quantifying PTX3 levels in BALF and plasma of non-neutropenic patients. Our findings demonstrate that both BALF and plasma PTX3 are significantly elevated in IPA patients, with optimal thresholds of 4.17 and 2.67 ng/ml, respectively. Notably, BALF PTX3 exhibited superior sensitivity compared to BALF GM. While PTX3 specificity remained lower than that of BALF GM, a combinatorial diagnostic strategy incorporating PTX3 and GM significantly enhanced overall diagnostic performance, particularly within the ICU setting. Collectively, these results establish PTX3 as a highly sensitive adjunctive biomarker for IPA, particularly valuable for rule-out purposes in high-risk, non-neutropenic populations.

The present study represents a substantial methodological and clinical advancement over our previous single-centre pilot work [15]. By shifting from a heterogenous cohort spanning invasive, subacute, and chronic manifestations to a rigorously defined prospective, multicenter cohort of 605 suspected IPA cases, we have refined the diagnostic thresholds for IPA. Li *et al*. reported lower diagnostic thresholds for PTX3 in plasma (2.3 ng/ml) and BALF (1.9 ng/ml) because their cohort encompassed both highly acute IPA patients and those with lower-inflammation chronic conditions [15]. Similarly, He *et al*. identified optimal PTX3 thresholds of 2.57 ng/ml in plasma and 2.16 ng/ml in BALF for diagnosing IPA in specifically within a COPD cohort [16]. The higher cutoff values observed in our comprehensive cohort likely reflect the intense inflammatory cascades and elevated PTX3 expression typical of an acute, critically ill non-neutropenic population. Furthermore, PTX3 levels were not significantly influenced by common baseline factors such as COPD, diabetes, or prior corticosteroid use, suggesting its diagnostic stability across diverse clinical backgrounds.

Previous studies have demonstrated that elevated PTX3 levels are associated with severe pneumonia and correlate with both prognosis and treatment response [18,19]. In our study, subgroup analysis of ICU patients further confirmed that PTX3 levels were higher in IPA patients compared to non-IPA patients. Although both plasma and BALF PTX3 exhibited diagnostic sensitivities exceeding 85%, the specificity remained relatively low, raising concerns about potential false-positive results. Notably, adjusting the cutoff value for plasma PTX3 to 11.81 ng/ml could enhance specificity to 83.87%, albeit at the expense of reduced sensitivity. These findings underscore the importance of balancing sensitivity and specificity when applying PTX3 as a diagnostic biomarker in clinical practice.

Compared with existing mycological biomarkers, PTX3 offers distinct diagnostic advantages for IPA detection. In our study, BALF PTX3 exhibited significantly higher sensitivity than the BALF GM assay, suggesting potential for earlier infection detection. The GM test detects galactomannan, a component of the *Aspergillus* cell wall, and offers high diagnostic specificity. However, its sensitivity can be compromised by factors such as prior antifungal therapy and host immune status, potentially leading to false-negative results [20,21]. To overcome these limitations, we employed a combined diagnostic approach, considering a positive result for either PTX3 or GM. This strategy improved the overall sensitivity to over 90%. Furthermore, combining plasma PTX3, GM, and *Asp* IgG further enhanced diagnostic sensitivity, facilitating early identification of IPA cases. In contrast, He *et al*. proposed a dual-positive strategy requiring concurrent PTX3 and GM positivity in COPD patients, achieving a specificity exceeding 90% but substantially reduced sensitivity, which is unfavourable for early IPA detection [16]. Notably, our findings revealed that both plasma and BALF PTX3 exhibited high negative predictive values (NPV), which were further enhanced when combined with conventional biomarkers. This suggests that in clinical practice, IPA can be reliably excluded when PTX3 levels fall below the diagnostic threshold, supporting its utility as a rule-out biomarker to avoid or discontinue unnecessary empirical antifungal therapy. Conversely, a positive PTX3 result should prompt parallel confirmatory testing (e.g. GM assays or cultures) rather than immediate therapeutic intervention. Additionally, pathogen-based examinations often fail to distinguish between *Aspergillus* infection and colonization. In our study, both plasma and BALF PTX3 levels were significantly higher in IPA patients compared to those with other forms of PA and *Aspergillus* colonization. This finding is consistent with that of Li *et al*., who reported a decreasing PTX3 levels from IPA to SAIA and CPA [15]. Similarly, Dima *et al*. suggested that BALF PTX3 could help distinguish between *Aspergillus* colonization and active infection in lung transplant recipients [22]. Therefore, these results highlight the potential of PTX3 as a differential biomarker across the spectrum of PA.

PTX3 is a non-specific inflammatory biomarker that may be upregulated in infectious diseases (e.g. *Streptococcus pneumoniae*, *Klebsiella pneumoniae*, *Pseudomonas aeruginosa*, *Acinetobacter baumannii*, COVID-19) [23–27] and non-infectious conditions such as cancers and autoimmune diseases [28–30]. In the present study, PTX3 levels were significantly higher in IPA patients, likely reflecting the intense inflammatory response and immune activation induced by *Aspergillus* infection. Importantly, PTX3 should be interpreted as an adjunctive biomarker in patients with a high clinical suspicion of IPA, based on host risk factors, clinical presentation, and radiological features. Its diagnostic application should be limited to such contexts, as PTX3 levels may also be elevated in other inflammatory conditions, including sepsis [31]. Moreover, plasma PTX3 levels in IPA patients were comparable to those observed in other fungal infections, primarily mucormycosis. This finding aligns with Dobiaš *et al*., who reported similar BALF PTX3 levels in IPA and invasive pulmonary mucormycosis [32], suggesting that PTX3 may function as a broad biomarker for invasive fungal infection (IFI) rather than an *Aspergillus*-specific marker. Due to limited sample sizes of non-*Aspergillus* fungal infections, comprehensive comparisons across diverse fungal pathogens were not feasible, and larger studies are warranted to validate these observations. In clinical practice, PTX3 elevation in patients with suspected IFI should be interpreted as indicative of a fungal infection signature, prompting further specific mycological investigations (e.g. BALF GM, PCR, or culture) to identify the causative pathogen, while leveraging its utility in differentiating IFI from bacterial infections and non-infectious pulmonary diseases.

This study has several limitations. First, the absence of standardized *Aspergillus* PCR protocols across participating centres precluded direct comparison with this highly sensitive diagnostic modality. However, subgroup analysis of BALF mNGS data demonstrated comparable diagnostic performance between mNGS and PTX3, suggesting that PTX3 remains a highly competitive biomarker for IPA diagnosis. Second, although we observed declining PTX3 levels following antifungal therapy, the small sample size precluded definitive analysis of treatment response kinetics. Future longitudinal studies are warranted to characterize PTX3 dynamics throughout the disease course and their correlation with therapeutic outcomes. Third, the limited representation of critically ill patients restricts the generalizability of our findings to ICU populations. Larger, prospective studies are needed to validate PTX3 diagnostic performance in this specific subgroup and to establish clinically relevant cutoff values for critically ill patients.

In summary, PTX3 in both plasma and BALF were significantly elevated in non-neutropenic IPA patients, supporting its potential as a promising biomarker for IPA diagnosis. Although PTX3 exhibited substantial diagnostic sensitivity, its specificity was relatively limited. Integrating PTX3 with established mycological biomarkers may further improve diagnostic accuracy.

## Supplementary Material

Supplementary file.docx
