# Supplementary material for: Diagnostic value of pentraxin 3 in plasma and bronchoalveolar lavage fluid for invasive pulmonary aspergillosis in non-neutropenic patients: a prospective multicenter clinical study
Source: Emerg Microbes Infect. 2026 Apr 29;15(1):2667562. doi: 10.1080/22221751.2026.2667562 (PMC13162536; doi:10.1080/22221751.2026.2667562)
Supplement: Supplementary file.docx [file TEMI_A_2667562_SM2852.docx]

**SUPPLEMENTAL MATERIALS**

**Manuscript Title:**

Diagnostic value of pentraxin 3 in plasma and bronchoalveolar lavage fluid for invasive pulmonary aspergillosis in non-neutropenic patients: a prospective multicenter clinical study

**Authors:**

Chao Sun^1,2*^, Huanhuan Zhong^3*^, Xiaomin Cai^2^, Min Cao^1^, Tingting Zhao^1^, Li Wang^4,5^, Chunlai Feng^6^, Wenkui Sun^7^, Cheng Chen^8^, Yanbin Chen^8^, Yujian Tao^9^, Jun Zhou^9^, Yueyan Ni^1^, Yu Gu^1^, Minhua Shi^3^, Xin Lu^10^, Guoer Ma^11^, Yuanqin Li^12^, Jiaxin Shi^9^, Yuchen Cai^2^, Mengyue Song^2^, Yuanyuan Li^2^, Yajie Lu^1^, Jinjin Zhong^2^, Yi Shi^2^, and Xin Su^1 #^

**Affiliations:**

^1^Department of Respiratory and Critical Medicine, Nanjing Drum Tower Hospital, Affiliated Hospital of Medical School, Nanjing University, Nanjing, China.

^2^Department of Respiratory and Critical Medicine, Jinling Hospital, Affiliated Hospital of Medical School, Nanjing University, Nanjing, China.

^3^Department of Respiratory and Critical Medicine, the Second Affiliated Hospital of Soochow University, Suzhou, China.

^4^Department of Respiratory and Critical Medicine, Nanjing First Hospital, Nanjing, China.

^5^Department of Respiratory and Critical Medicine, Jiangsu Province Second Chinese Medicine Hospital, Nanjing, China.

^6^Department of Respiratory and Critical Medicine, Changzhou First People's Hospital, Changzhou, China.

^7^Department of Respiratory and Critical Medicine, Jiangsu Province Hospital, Nanjing, China.

^8^Department of Respiratory and Critical Medicine, the First Affiliated Hospital of Soochow University, Suzhou, China.

^9^Department of Respiratory and Critical Medicine, Affiliated Hospital of Yangzhou University, Yangzhou, China.

^10^Department of Respiratory and Critical Medicine, Nanjing Jiangning Hospital, Nanjing, China.

^11^Department of Respiratory and Critical Medicine, Affiliated Hospital of Jiangsu University, Zhenjiang, China.

^12^Department of Respiratory and Critical Medicine, Affiliated Hospital of Xuzhou Medical University, Xuzhou, China.

*These authors contributed equally to this work.

**^#^ Corresponding author:**

Xin Su, Department of Respiratory and Critical Care Medicine, Nanjing Drum Tower Hospital, The Affiliated Hospital of Nanjing University Medical School, Nanjing 210008, China.

Email address: suxinjs@163.com (X. Su)

**Supplementary methods**

**Diagnostic criteria of IPA**

The grading diagnostic criteria of IPA referred to the 2020 guidelines from the European Organization for Research and Treatment of Cancer and the Mycoses Study Group Education and Research Consortium (EORTC/MSGERC)^[1]^, and the 2024 consensus definitions from ESGCIP, EFISG, ESICM, ECMM, MSGERC, ISAC, and ISHAM^[2]^. “Proven IPA” requires histopathologic confirmation of *Aspergillus* hyphae in a normally sterile site or the lung obtained through biopsy or needle aspiration. “Probable IPA” needs a comprehensive evaluation of host factors, symptoms, clinical criteria, and mycological evidence. Host factors pertain to underlying chronic pulmonary or extrapulmonary diseases, such as chronic obstructive pulmonary disease (COPD), diabetes, or admission to an intensive care unit (ICU). Clinical symptoms are characterized by respiratory manifestations, including cough, sputum production, dyspnea, or chest pain, which persist despite broad-spectrum antibiotic therapy. The clinical criterion is chest CT scan showing infiltrates or cavitation not attributable to other causes. Mycological evidence included one or more of the following: a serum or plasma GM level greater than 0.5 optical density index (ODI), a BALF GM level at least 1.0 ODI, or positive *Aspergillus* culture from BALF, bronchial brush, or aspirate, and at least two positive *Aspergillus* PCR tests. “Possible IPA” met host factors, clinical symptoms and clinical criterion.

**References:**

[1] Donnelly JP, Chen SC, Kauffman CA, Steinbach WJ, Baddley JW, Verweij PE, et al. Revision and Update of the Consensus Definitions of Invasive Fungal Disease From the European Organization for Research and Treatment of Cancer and the Mycoses Study Group Education and Research Consortium. Clin Infect Dis. 2020;71(6):1367-76.

[1] Bassetti M, Giacobbe DR, Agvald-Ohman C, Akova M, Alastruey-Izquierdo A, Arikan-Akdagli S, et al. Invasive Fungal Diseases in Adult Patients in Intensive Care Unit (FUNDICU): 2024 consensus definitions from ESGCIP, EFISG, ESICM, ECMM, MSGERC, ISAC, and ISHAM. Intensive Care Med. 2024;50(4):502-15.

**Supplementary figures:**


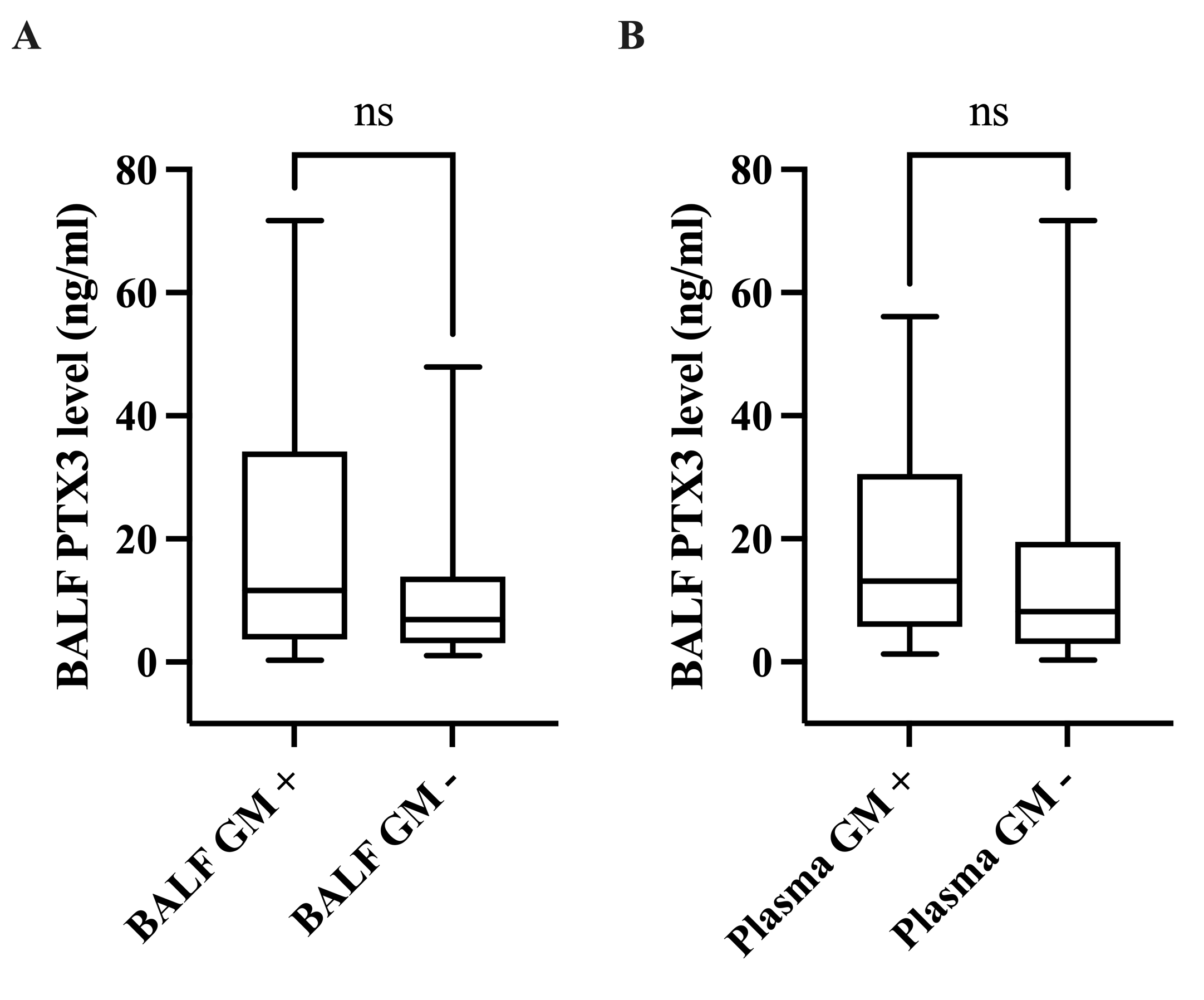


**Figure S1** Comparison of PTX3 levels in BALF between GM test (BALF and plasma) positive and negative IPA patients. (A) BALF PTX3 levels among IPA patients with positive BALF GM test results or negative. BALF GM level greater than 1.0 optical density index is considered positive. (B) BALF PTX3 levels among IPA patients with positive plasma GM test results or negative. Plasma GM level greater than 0.5 optical density index is considered positive. ns: P＞0.05.

PTX3, pentraxin 3; IPA, invasive pulmonary aspergillosis; BALF, bronchoalveolar lavage fluid; GM, galactomannan.


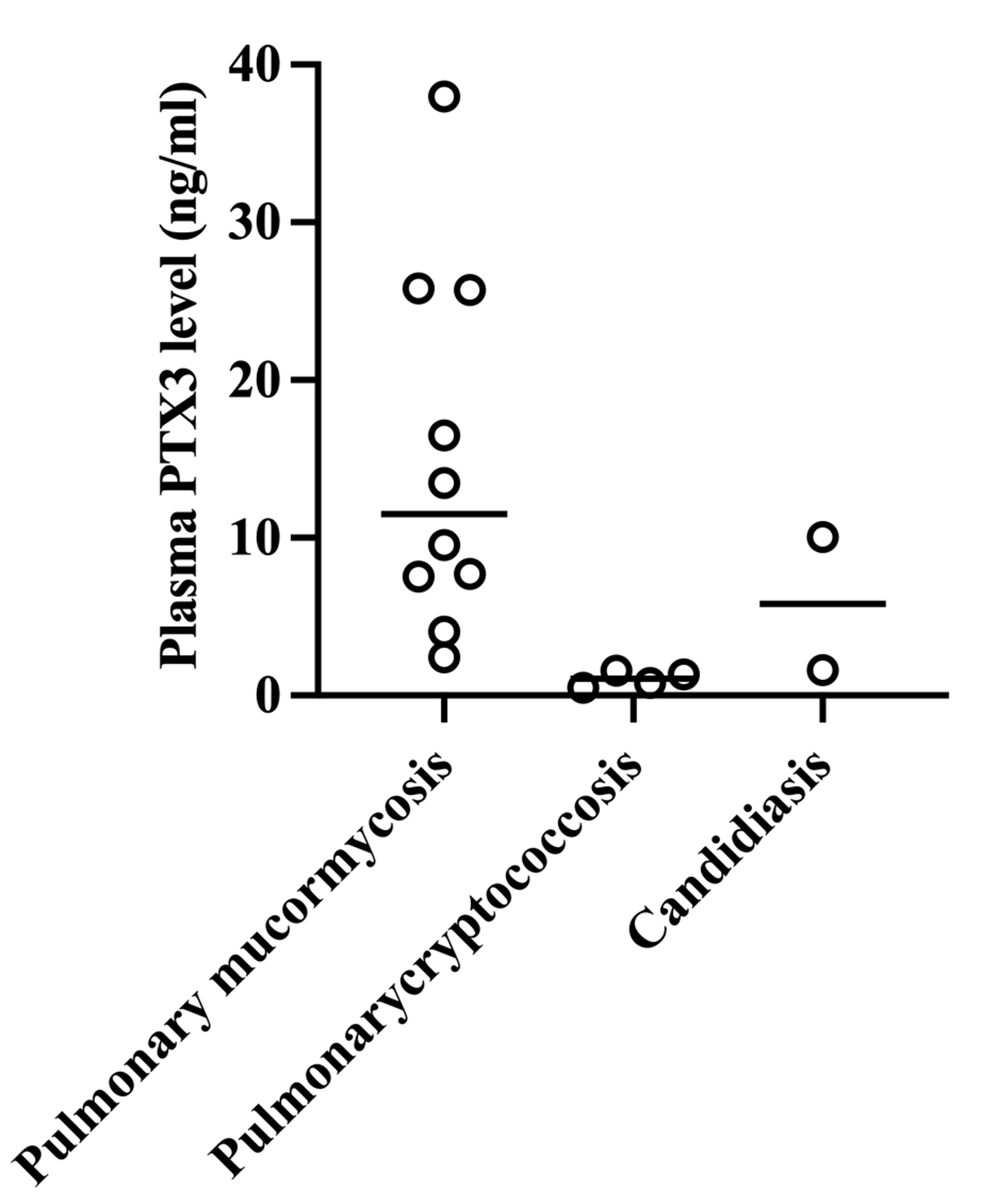


**Figure S2** Plasma PTX3 levels in different fungal infections. PTX3, pentraxin 3.


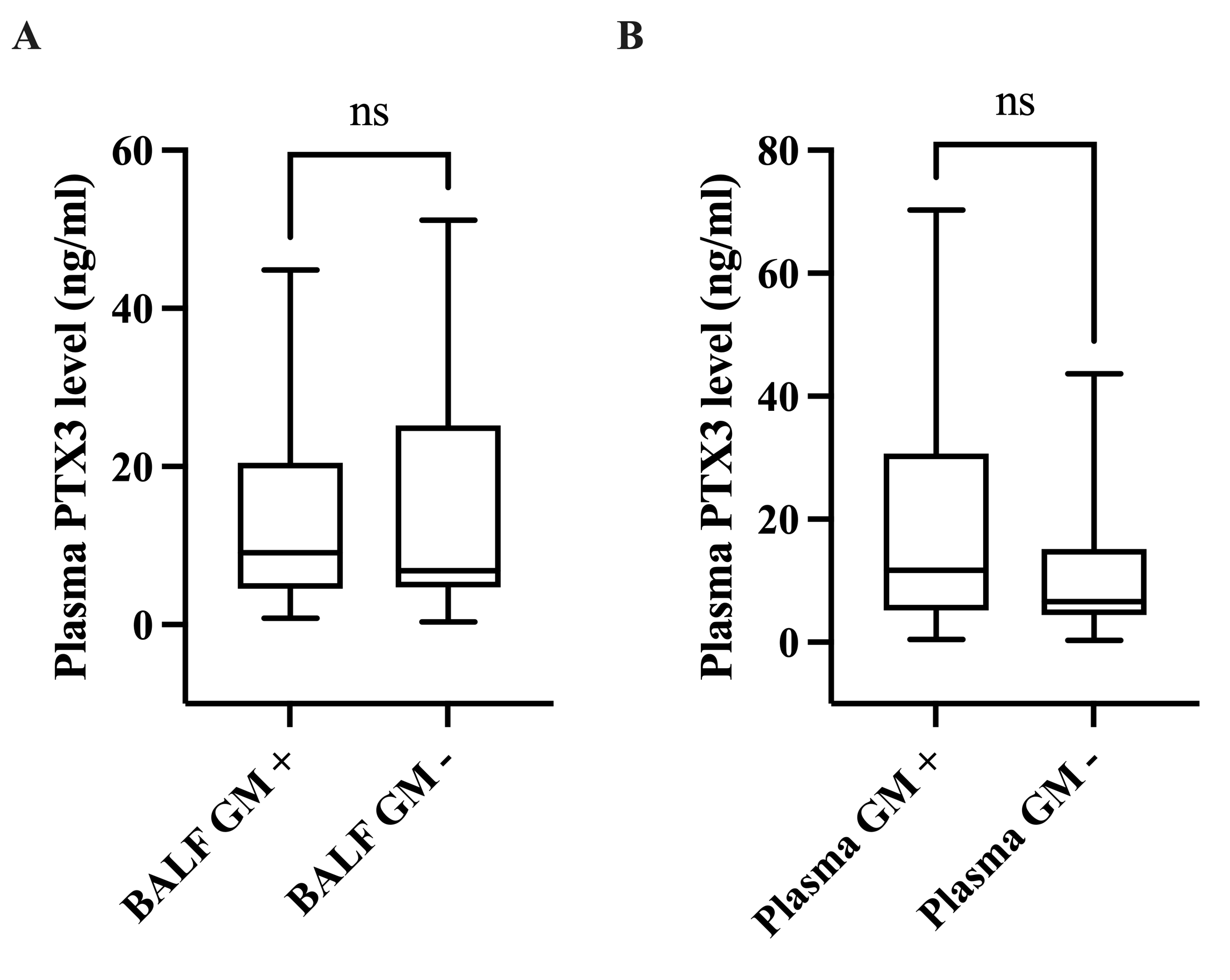


**Figure S3** Comparison of PTX3 levels in plasma between GM test (BALF and plasma) positive and negative IPA patients. (A) Plasma PTX3 levels among IPA patients with positive BALF GM test results or negative. BALF GM level greater than 1.0 optical density index is considered positive. (B) Plasma PTX3 levels among IPA patients with positive plasma GM test results or negative. Plasma GM level greater than 0.5 optical density index is considered positive. ns: P＞0.05.

PTX3, pentraxin 3; IPA, invasive pulmonary aspergillosis; BALF, bronchoalveolar lavage fluid; GM, galactomannan.


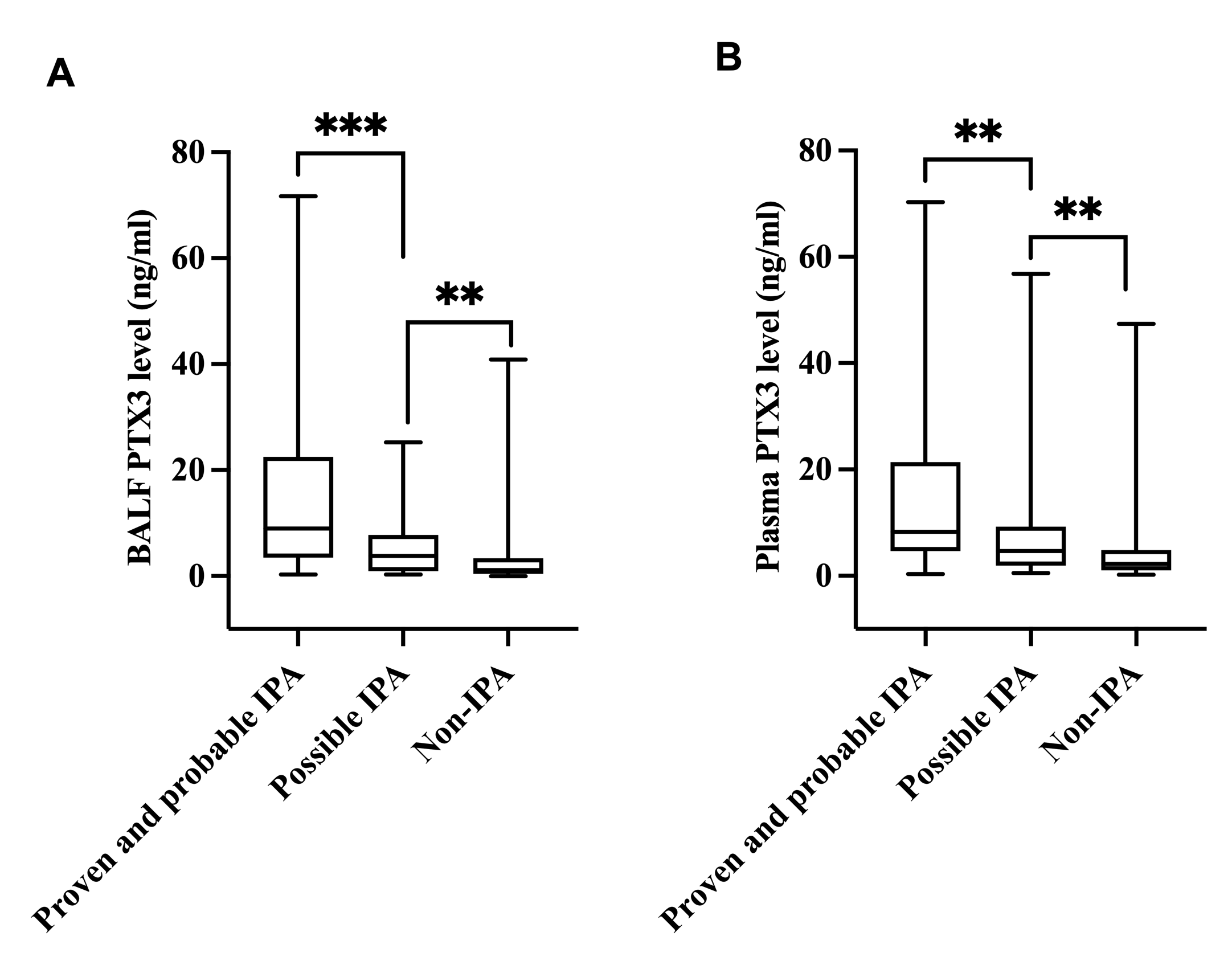


**Figure S4** PTX3 levels across different diagnostic categories of IPA.

(A) BALF PTX3 levels in proven+probable IPA (n=97), possible IPA (n=26), and non-IPA (n=290) patients. (B) Plasma PTX3 levels in proven+probable IPA (n=122), possible IPA (n=38), and non-IPA (n=288) patients. **: P＜0.01; ***: P＜0.001.

PTX3, pentraxin 3; IPA, invasive pulmonary aspergillosis; BALF, bronchoalveolar lavage fluid.


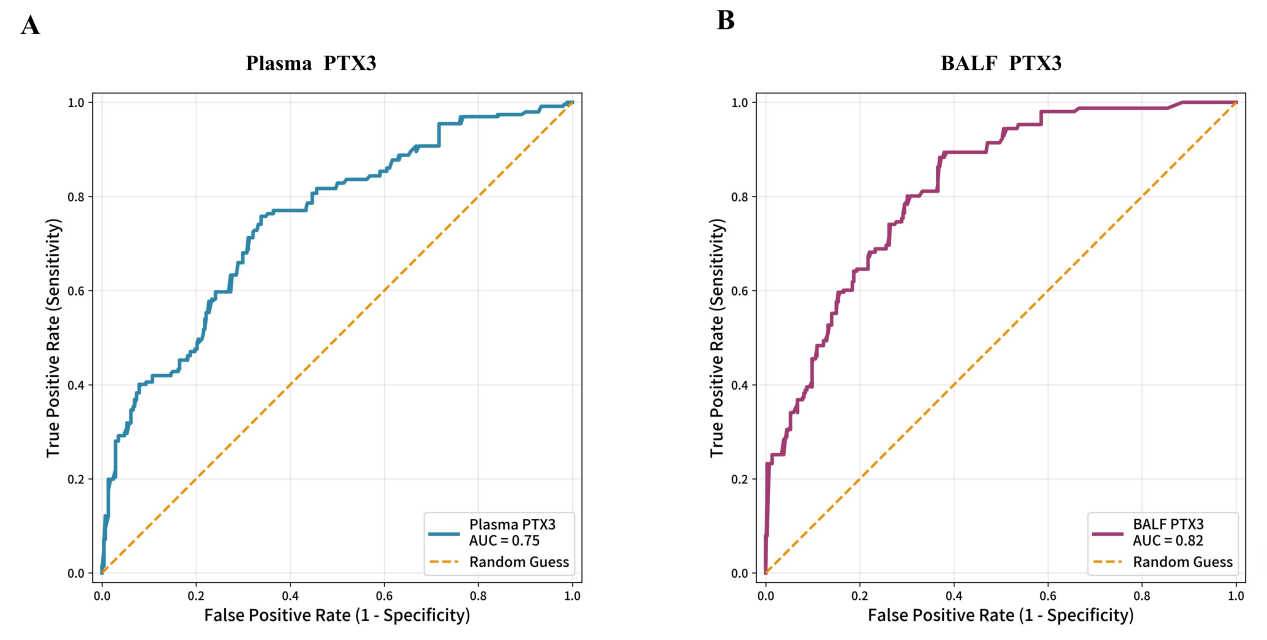


**Figure S5** IPTW-weighted ROC curves of PTX3 for diagnosing IPA.

Propensity scores were estimated using a multivariable logistic regression model including age, ICU admission, COPD, diabetes, and coexisting bacterial infection to balance potential confounders between IPA cases and non-IPA controls.

1. Plasma PTX3, AUC 0.75 (95% CI: 0.69-0.81). (B) BALF PTX3, AUC 0.82 (95% CI: 0.76-0.87).

IPTW, inverse probability of treatment weighting; ROC, receiver operating characteristic; PTX3, pentraxin 3; IPA, invasive pulmonary aspergillosis; AUC, area under the curve; CI, confidence interval; BALF, bronchoalveolar lavage fluid; ICU, intensive care unit.


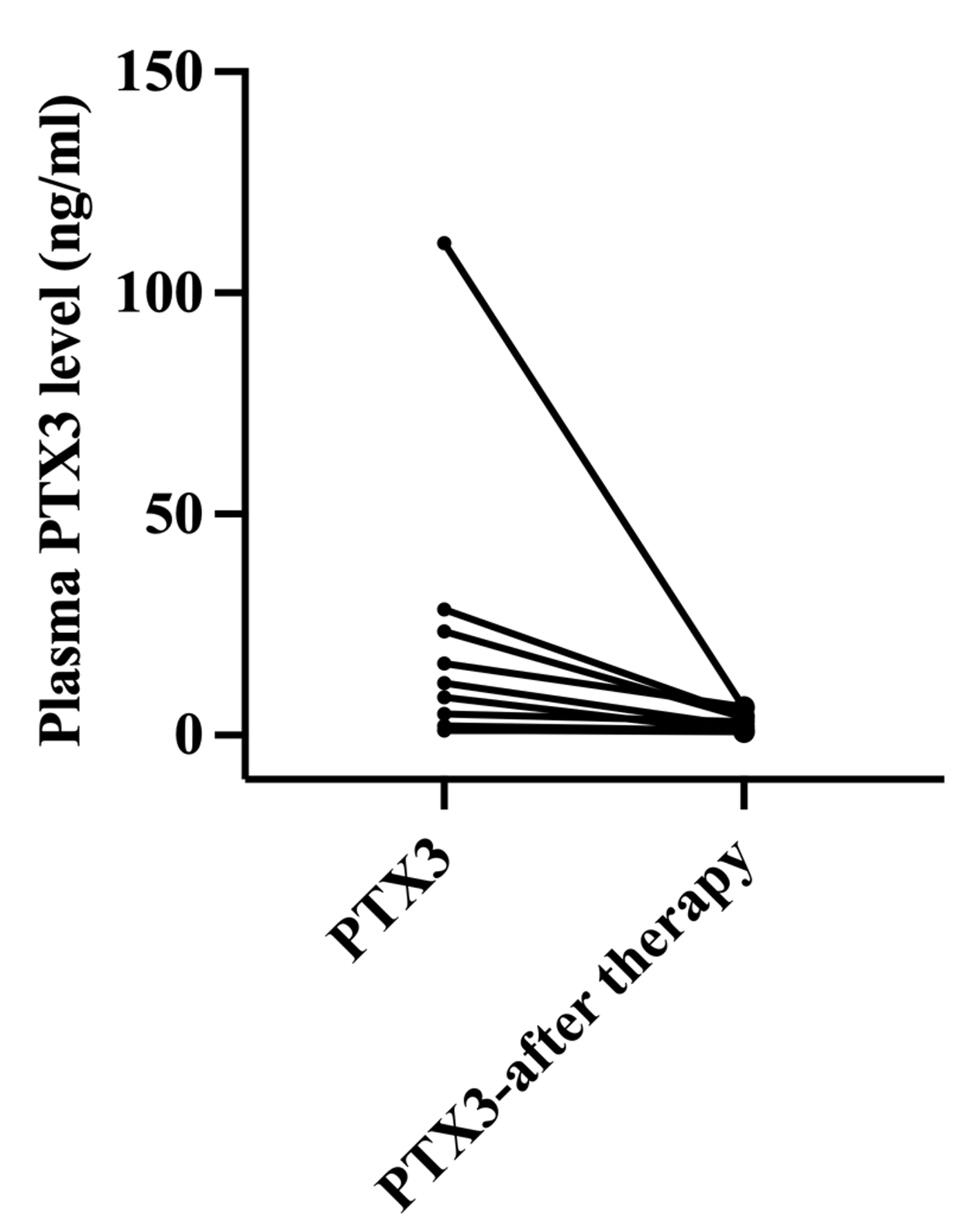


**Figure S6** Changes in plasma PTX3 levels in IPA patients before and after antifungal treatment. P = 0.002.

PTX3, pentraxin 3; IPA, invasive pulmonary aspergillosis.

**Supplementary tables:**

**Table S1**: BALF and plasma PTX3 levels in IPA and non-IPA subgroups.

| **Group** | **BALF PTX3 (ng/ml), media [IQR]** | **P value^ᵃ^** | **Plasma PTX3 (ng/ml), media [IQR]** | **P value^ᵃ^** |
| --- | --- | --- | --- | --- |
| **IPA** | 8.98 [3.47–22.53] | - | 8.29 [4.68–21.41] | - |
| **Non-IPA** | 1.12 [0.46–3.35] | < 0.0001 | 2.60 [1.35–5.02] | < 0.0001 |
| CAP | 1.26 [0.46–4.48] | < 0.0001 | 2.91 [1.62–5.41] | < 0.0001 |
| TB | 1.14 [0.44–3.04] | < 0.0001 | 2.65 [1.47–4.28] | < 0.0001 |
| Non-infectious diseases | 0.48 [0.29–1.00] | < 0.0001 | 2.09 [1.00–3.70] | < 0.0001 |
| Other fungal infections | 0.74 [0.35–1.77] | < 0.0001 | 7.62 [1.59–15.75] | 0.175 |
| Other types of PA | 1.59 [0.53–2.88] | < 0.0001 | 1.87 [0.86–3.50] | < 0.0001 |

BALF, bronchoalveolar lavage fluid; PTX3, pentraxin 3; IPA, invasive pulmonary aspergillosis; IQR, interquartile range; CAP, community-acquired pneumonia; TB, tuberculosis; PA, pulmonary aspergillosis.

^ᵃ^: P value compared with the IPA group.

**Table S2**：Sensitivity analysis of the diagnostic performance of PTX3 in BALF and plasma.

|  | **Cutoff value (ng/ml)** | **AUC (95% CI)** | **Sensitivity% (95% CI)** | **Specificity% (95% CI)** | **PPV%**  **(95% CI)** | **NPV%**  **(95% CI)** |
| --- | --- | --- | --- | --- | --- | --- |
| **BALF PTX3** | | | | | | |
| Proven/Probable IPA vs Non-IPA | 2.67 | 0.84 (0.80-0.89) | 85.57 (77.22-91.20) | 71.72 (66.28-76.60) | 50.30 (42.75-57.83) | 93.69 (89.70-96.21) |
| SA1^b^: +Possible vs Non-IPA | 2.67 | 0.81 (0.77-0.85) | 80.49 (72.61-86.52) | 71.72 (66.28-76.60) | 54.70 (47.40-61.80) | 89.66 (85.03-92.97) |
| SA 2^c^: vs Possible+Non-IPA | 2.67 | 0.83 (0.79-0.88) | 85.57 (77.22-91.20) | 68.99 (63.69-73.83) | 45.86 (38.73-53.15) | 93.97 (90.07-96.39) |
| **Plasma PTX3** | | | | | | |
| Proven/Probable IPA vs Non-IPA | 4.17 | 0.80 (0.75-0.85) | 79.51 (71.50-85.72) | 69.79 (64.26-74.81) | 52.72 (45.52-59.80) | 88.94 (84.18-92.39) |
| SA1^b^: +Possible vs Non-IPA | 4.16 | 0.76 (0.71-0.81) | 73.13 (65.77-79.39) | 69.79 (64.26-74.81) | 57.35 (50.47-63.96) | 82.38 (77.08-86.66) |
| SA 2^c^: vs Possible+Non-IPA | 4.16 | 0.78 (0.73-0.83) | 79.51 (71.50-85.72) | 67.48 (62.22-72.34) | 47.78 (41.00-54.65) | 89.80 (85.33-93.01) |

BALF, bronchoalveolar lavage fluid; PTX3, pentraxin 3; AUC, area under the receiver operating characteristic curve; PPV, positive predictive value; NPV, negative predictive value; CI, confidence interval; IPA, invasive pulmonary aspergillosis; SA, subgroup analysis.

^b^: SA1 expanded the IPA group by including possible IPA cases.

^c^: SA2 expanded the non-IPA group by including possible IPA cases.

**Table S3**：Multivariable logistic regression analysis evaluating PTX3 as an independent predictor of IPA.

| **Variable** | **P value** | **OR (95% CI)** |
| --- | --- | --- |
| Age, years | 0.353 | 1.016 (0.983-1.049) |
| ICU admission | 0.076 | 2.078 (0.926-4.660) |
| COPD | 0.015 | 3.082 (1.243-7.642) |
| Diabetes | 0.708 | 0.849 (0.360-2.002) |
| Plasma PTX3, per 1 ng/ml increase | <0.001 | 1.074 (1.036-1.113) |
| BALF PTX3, per 1 ng/ml increase | <0.001 | 1.111 (1.065-1.159) |
| Coexisting bacterial infection | 0.064 | 0.472 (0.214-1.044) |

BALF, bronchoalveolar lavage fluid; PTX3, pentraxin 3; IPA, invasive pulmonary aspergillosis; ICU, intensive care unit; COPD, chronic obstructive pulmonary disease; OR, odds ratio; CI, confidence interval.

**Table S4**: Comparison of the diagnostic performance of PTX3, GM, mNGS, and *Asp* IgG in ICU-admitted IPA patients.

| **Biomarker** | **Cutoff value** | **Sensitivity% (95% CI)** | **Specificity% (95% CI)** | **PPV%**  **(95% CI)** | **NPV%**  **(95% CI)** |
| --- | --- | --- | --- | --- | --- |
| **BALF** | | | | | |
| PTX3 | 2.67 ng/ml | 95.56 (85.17-98.77) | 54.00 (40.40-67.03) | 65.15 (53.11-75.52) | 93.10 (78.04-98.09) |
| GM | 1.0 ODI | 79.69 (68.29-87.73) | 91.23 (81.05-96.19) | 91.07 (80.74-96.13) | 80.00 (68.73-87.92) |
| mNGS | **Positive detection** | 81.25 (64.69-91.11) | 93.10 (78.04-98.09) | 92.86 (77.35-98.02) | 81.82 (65.61-91.39) |
| PTX3/GM | -^d^ | 97.62 (87.68-99.58) | 48.89 (34.96-63.00) | 64.06 (51.82-74.71) | 96.65 (79.01-99.23) |
| **Plasma** | | | | | |
| PTX3 | 11.81 ng/ml | 53.03 (41.16-64.57) | 83.87 (72.79-91.00) | 77.78 (63.73-87.46) | 62.65 (51.90-72.28) |
| GM | 0.5 ODI | 40.58 (29.79-52.36) | 94.20 (86.02-97.72) | 87.50 (71.93-95.03) | 61.32 (51.81-70.04) |
| *Asp* IgG | 80 AU/ml | 61.54 (47.96-73.53) | 58.62 (45.80-70.37) | 57.14 (44.13-69.23) | 62.96 (49.63-74.58) |
| PTX3/GM | -^d^ | 73.44 (61.52-82.70) | 77.59 (65.34-86.41) | 78.33 (66.38-86.88) | 72.58 (60.41-82.12) |
| PTX3/GM/  *Asp* IgG | -^d^ | 86.27 (74.28-93.19) | 47.17 (34.38-60.34) | 61.11 (49.56-71.53) | 78.12 (61.25-88.98) |

BALF, bronchoalveolar lavage fluid; PTX3, pentraxin 3; GM, galactomannan; mNGS, metagenomic next-generation sequencing; ODI, optical density index; *Asp* IgG, *Aspergillus*-specific IgG antibody; ICU, intensive care unit; IPA, invasive pulmonary aspergillosis; PPV, positive predictive value; NPV, negative predictive value; CI, confidence interval.

^d^: A parallel testing strategy was used for combined biomarkers, whereby a positive result was defined as positivity in at least one component of the combination.
